# Heterotrimeric G Protein-Regulated Ca^2+^ Influx and PIN2 Asymmetric Distribution Are Involved in *Arabidopsis thaliana* Roots' Avoidance Response to Extracellular ATP

**DOI:** 10.3389/fpls.2017.01522

**Published:** 2017-09-01

**Authors:** Ruojia Zhu, Xiaoxia Dong, Weiwei Hao, Wei Gao, Wenzhu Zhang, Shuyan Xia, Ting Liu, Zhonglin Shang

**Affiliations:** Hebei Key Laboratory of Molecular and Cellular Biology, College of Life Sciences, Hebei Normal University Shijiazhuang, China

**Keywords:** extracellular ATP, heterotrimeric G protein, auxin, calcium, *Arabidopsis thaliana* L.

## Abstract

Extracellular ATP (eATP) has been reported to be involved in plant growth as a primary messenger in the apoplast. Here, roots of *Arabidopsis thaliana* seedlings growing in jointed medium bent upon contact with ATP-containing medium to keep away from eATP, showing a marked avoidance response. Roots responded similarly to ADP and bz-ATP but did not respond to AMP and GTP. The eATP avoidance response was reduced in loss-of-function mutants of heterotrimeric G protein α subunit (Gα) (*gpa1-1* and *gpa1-2*) and enhanced in Gα-over-expression (OE) lines (*wG*α and *cG*α). Ethylenebis(oxyethylenenitrilo) tetraacetic acid (EGTA) and Gd^3+^ remarkably suppressed eATP-induced root bending. ATP-stimulated Ca^2+^ influx was impaired in Gα null mutants and increased in its OE lines. DR5-GFP and PIN2 were asymmetrically distributed in ATP-stimulated root tips, this effect was strongly suppressed by EGTA and diminished in Gα null mutants. In addition, some eATP-induced genes' expression was also impaired in Gα null mutants. Based on these results, we propose that heterotrimeric Gα-regulated Ca^2+^ influx and PIN2 distribution may be key signaling events in eATP sensing and avoidance response in *Arabidopsis thaliana* roots.

## Introduction

Recent discoveries revealed that adenosine triphosphate (ATP) can be found not only in the cytoplasm, but also in the extracellular matrix of plant cells (Kim et al., 2006). ATP is prevalent in the apoplast of various plant species (Kim et al., 2006) and can be released through secretory vesicles (Kim et al., 2006; Wu et al., 2008) or plasma membrane (PM) injury (Song et al., 2006). Various stimuli, including touch (Jeter et al., 2004; Weerasinghe et al., 2009; Vanegas et al., 2015), osmotic stress (Jeter et al., 2004), chitin mixture (Kim et al., 2006), polysaccharide elicitors (Wu et al., 2008), and hypertonic stress (Kim et al., 2009), have been shown to induce ATP release.

Extracellular ATP (eATP) is involved in cell viability maintenance (Chivasa et al., 2005; Sun et al., 2012a), growth rate regulation (Kim et al., 2006; Wolf et al., 2007; Wu, J. et al., 2007; Riewe et al., 2008; Clark et al., 2010; Tonon et al., 2010), pollen germination (Steinebrunner et al., 2003; Reichler et al., 2009), stomatal movement (Clark et al., 2011; Hao et al., 2012; Wang et al., 2014), and biotic & abiotic stress resistance (Thomas et al., 2000; Windsor et al., 2003; Chivasa et al., 2009; Kim et al., 2009; Sun et al., 2012b; Deng et al., 2015). It is therefore well accepted that eATP acts as a multi-functional apoplast signaling molecule in plants.

The elucidation of eATP signal transduction has been an active area of research over the last decade. Early pharmacological studies indirectly indicated that receptors similar to the animal eATP receptor may exist in plant cells (Demidchik et al., 2003; Chivasa et al., 2005; Song et al., 2006; Sun et al., 2012a). Recent studies showed that the *Arabidopsis thaliana* lectin receptor kinase (LecRK-1.9) is an eATP receptor and subsequently annotated it the P2K receptor (Choi et al., 2014a; Balague et al., 2016). Signal transduction components in the PM, including heterotrimeric G proteins (Weerasinghe et al., 2009; Tanaka et al., 2010; Hao et al., 2012), Ca^2+^ channels (Demidchik et al., 2009; Wang et al., 2014) and NADPH oxidase (Song et al., 2006; Demidchik et al., 2009; Tonon et al., 2010; Hao et al., 2012; Wang et al., 2014), have been shown to be involved in the generation of eATP-induced second messengers. These second messengers, which include cytoplasmic Ca^2+^ (Demidchik et al., 2003; Jeter et al., 2004; Tanaka et al., 2010; Hao et al., 2012), reactive oxygen species (Kim et al., 2006; Song et al., 2006; Demidchik et al., 2009; Tonon et al., 2010; Hao et al., 2012; Sun et al., 2012b; Lim et al., 2014; Wang et al., 2014), and nitric oxide (Foresi et al., 2007; Wu and Wu, 2008; Reichler et al., 2009; Tonon et al., 2010; Clark et al., 2011), play essential roles in eATP-induced physiological reactions. The expression of functional genes induced by these second messengers may eventually lead to eATP-regulated physiological reactions (Chivasa et al., 2011; Lim et al., 2014).

Furthermore, several plant hormones, such as auxin (Tang et al., 2003; Liu et al., 2012), ethylene (Jeter et al., 2004; Clark et al., 2010), and salicylic acid (Chivasa et al., 2009), have been shown to participate in eATP-regulated physiological functions, suggesting that eATP may regulate physiological processes by crosstalk with these hormones.

As an important PM signal transducer, heterotrimeric G protein is involved in plant growth and development (Urano and Jones, 2014; Stateczny et al., 2016; Urano et al., 2016), and the plant hormones auxin, GA, ethylene and ABA have been suggested to act via heterotrimeric G proteins (Ashikari et al., 1999; Ullah et al., 2003; Pandey et al., 2006; Jin et al., 2013; Ge et al., 2015). Interestingly, heterotrimeric G protein is also involved in eATP release (Weerasinghe et al., 2009), eATP-induced cytosolic Ca^2+^ elevation (Tanaka et al., 2010), and eATP-promoted stomatal movements (Hao et al., 2012). The involvement and signaling role of heterotrimeric G protein in the signal transduction of eATP regulated physiological responses need to be clarified.

Extracellular ATP (eATP) has been shown to inhibit root growth and promote the generation of adventitious roots. High concentrations of eATP suppressed the growth of *Arabidopsis* roots or even induced root curling (Tang et al., 2003; Liu et al., 2012). In tilted medium, root skewing was also promoted by eATP (Yang et al., 2015). Nevertheless, the physiological significance and signal transduction of the eATP-regulated re-orientation of root growth remain unclear. Here, a jointed medium was used to verify the root tip response to eATP and the role of heterotrimeric Gα in this process.

## Materials and methods

### Plant material

*Arabidopsis thaliana* L. wild type and null mutants of α-, β- or γ-subunit of heterotrimeric G protein were used as material. Two ecotypes of wild type, Wassilewskija (WS) and Columbia-0 (Col-0) were used. Gα null mutants (*gpa1-1* and *gpa1-2*) seeds were a gift from Professor Alan Jones, North Carolina University at Chapel Hill, Chapel Hill, NC, USA. Gα overexpression lines (*wG*α and *cG*α) seeds were a gift from Professor Xingwang Deng, Yale University, New Haven, CT, USA. Gβ null mutants (*agb1-1* and *agb1-2*), Gγ null mutants (*agg1-1* and *agg1-2*), and PIN2 null mutant (*pin2*, salk-122916) seeds were obtained from *Arabidopsis* Biological Research Center, the Ohio State University. P2K receptor DORN1 null mutants (*dorn1-1* and *dorn1-3*) seeds were a gift from Dr. Julia Davies, Department of Plant Science, University of Cambridge, Cambridge, UK. All seeds were identified to confirm the homozygous mutation of corresponding gene.

### Root growth measurement

*Arabidopsis thaliana* seeds were surface-sterilized with 70% ethanol for 2 min followed by 5% sodium hypochlorite for 5 min. After two washes with sterilized water, seeds were sown on the surface of solid 1/2 MS medium (containing 0.8% phytagel) in square culture dishes. The culture dishes were stored at 4°C for 2 days and then were vertically cultured at 22°C and 130 μmol/m^2^·s illumination with 16/8 light/dark cycle.

To make the jointed medium, a solution of 1/2 MS salt and 0.8% phytagel was sterilized and poured into 10 × 10 cm square culture dishes, with each dish containing 50 mL liquid medium. After the medium solidified, the medium was cut with a sterilized blade along the midline of the culture dish, and half of the medium was removed. The interspace was then re-filled with 25 mL sterilized 1/2 MS medium containing ATP or other nucleotides (GTP, bz-ATP, ADP, and AMP). Nucleotides were dissolved with 1/2 MS solution to make a stock solution. The pH of stock solution was adjusted to 6.0 with Tris. The stock solution was filtered with a sterilized filter (SLGP033RB, 0.22 μm, Millipore, USA) and mixed with sterilized 1/2 MS medium which was cooled down to 50°C (to prevent possible degradation of added nucleotides) and poured into the interspace in the culture dish. After solidification, the refilled medium was level with the original medium. The concentration of nucleotides in medium was designed according to Tang et al. (2003) and Liu et al. (2012).

*Arabidopsis* seedlings which were growing in 1/2 MS medium for 4 days were transplanted onto the untreated part of the jointed medium, with the root tip toward the refilled medium and 0.3–0.5 cm from the joint line. The culture dishes were placed vertically, with the untreated part on top and the refilled nucleotide- containing medium at the bottom so the root will grow downward toward the refilled part where it will encounter different reagents in the medium.

To measure the root growth rate and curvature, photos of seedlings were captured using an optical scanner and then analyzed using Image J software. The curvature between the root growth orientation and the vertical line was measured as the root curvature. In each experiment, at least 30 seedlings were measured, and the mean value was calculated from three replicates. Data were statistically analyzed using Microsoft Excel software. The significance of differences between control and treatment groups was determined by Student's *t*-test.

### Electrophysiology

The Ca^2+^ influx channel activity was detected according to Demidchik et al. (2009). The bath solution consisted of 20 mM CaCl_2_, 0.1 mM KCl and 2 mM Tris, and the pH was adjusted to 6.0 with MES. The pipette solution consisted of 0.5 mM CaCl_2_, 5 mM Ca(OH)_2_, and 10 mM BAPTA (1,2-bis(2-aminophenoxy)ethane-N,N,N,N- tetra-acetic acid), 2 mM Mg·ATP, 0.5 mM Tris·ATP and 5 mM Tris, and the pH was adjusted to 7.0 with MES. The osmotic potentials of the bath and pipette solutions were adjusted to 300 mOsM with D-sorbitol.

To separate root protoplasts, root tips (approximately 5 mm long) from 4-day old seedlings were cut into short sections and digested at 25°C in bath solution containing 1% cellulase (Onozuka R-10, Yakult, Japan) and 0.5% pectinase (Onozuka Y-23, Yakult, Japan). After 1.5 h, the digested solution was filtered through a 100 μm diameter nylon mesh. After centrifugation and two washes with bath solution, the protoplasts were stored at 0°C.

The patch-clamp procedures were carried out according to Demidchik et al. (2009) and Wang et al. (2014). Whole cell step- and ramp-voltage clamping were performed. The current-voltage relationships were recorded within 2 min. To plot the I-V relationship curve, data from 6 protoplasts were calculated to get the mean value.

### Confocal laser scanning microscopy

To detect abundance and distribution of auxin and auxin efflux transporter, published *DR5-GFP, PIN1-GFP, PIN2-GFP, PIN3-GFP*, and *PIN7-GFP* transgenic lines (Friml et al., 2003) were used as material. DR5 is an auxin response promoter, which was widely used to drive reporter genes (*GUS* or *GFP*). PIN (PIN-FORMED proteins) are auxin efflux transporters in PM of plant cells. Each *PINx-GFP* is *PINx promoter-PINx-GFP* fusion genes. Seeds were purchased from *Arabidopsis* Biological Resource Center, the Ohio State University. *DR5-GFP* or *PIN2-GFP* was transformed into WS and Gα null mutants (*gpa1-1, gpa1-2*) by crossing with the respective lines above. Expression of the transformed genes was detected by PCR.

Seeds were germinated on solid 1/2 MS medium, and 4-day-old seedlings were transplanted onto jointed medium. After a period of time, seedlings were placed onto the stage of a microscope equipped with a laser confocal scanning system (LSM 710, Zeiss, Germany). The excitation/emission wavelengths were 488 and 510 nm, respectively. Captured images were then processed with Confocal Assistant software and further edited with Adobe Photoshop 7.0 software.

To measure fluorescence intensity in root tip cells, 0.5 mm long root tip was delineated as area of interest. To get the ratio of fluorescence intensity, cells on the left-side and right-side of root axis were delineated as area of interest. Fluorescence intensity in root cells was measured with Image J software and then calculated to get mean value. The significance of differences between control and treatment groups was determined by Student's *t*-test.

### Gene expression analysis

Wild type and heterotrimeric Gα null mutants (*gpa1-1, gpa1-2*) were used as material. Seedlings were germinated and grown on 1/2 MS medium for 5–6 days and then transplanted onto 0.3 mM ATP-containing jointed medium. To ensure that the root tip contacted eATP, 0.5 cm of root tip was placed onto the ATP-containing lower part and the rest of seedling onto the upper part. The culture dishes were then vertically placed. After 30 or 60 min, seedlings were taken out and 1 cm long root tips were processed for RNA extraction. Total RNA was isolated from 0.1 g root tissue using the RNAiso plus reagent (TaKaRa BIO., Japan), and 2 μg of total RNA was reverse-transcribed to cDNA using M-MLV reverse transcriptase (Promega, USA). Hybridization was performed according to the manufacturer's instruction (GeneChip® 3′ IVT PLUS Reagent Kit, Affymetrix). Synthesize biotin-PE (P-phycoerythrin) labeled cRNA by *in vitro* transcription. Incubate the labeled cRNA with hybridization master mix and then inject into the array. After hybridization and washing, GeneChip arrays were scanned on an Affymetrix probe array scanner (Affymetrix Inc., USA). Data were analyzed using the Partek software (Affymetrix Inc., USA). Raw data are available at https://www.ncbi.nlm.nih.gov/geo/info/linking.html., account number GSE102691.

To confirm the expression of specific genes, cDNA was amplified by real-time PCR reactions using SYBR Premix Ex TapTM (TaKaRa BIO., Japan) with gene-specific primers. *ACTIN2* was used as a reference gene. Delta delta CT approach was used to quantify gene expression levels. qRT-PCR reactions were performed on an ABI 7500 sequence detection system (Applied Biosystems, USA). All PCR primers are listed in Table S1.

## Results

### The avoidance response of *Arabidopsis thaliana* primary roots to eATP

**Figure 1:**
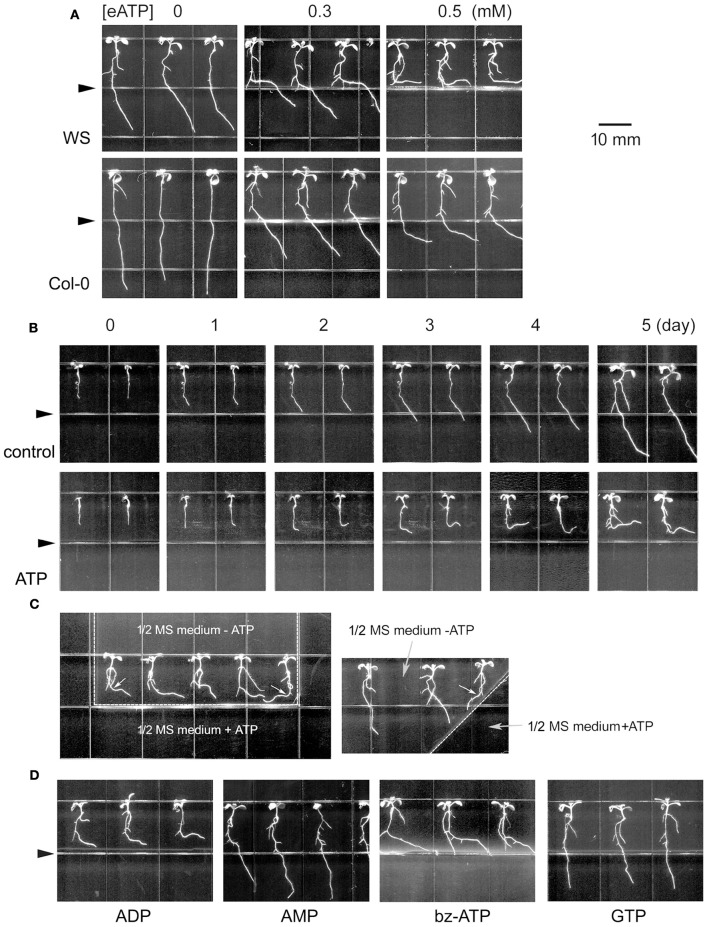
Extracellular ATP avoidance response of *Arabidopsis* roots. **(A)** Seedlings of *Arabidopsis thaliana* (WS and Col-0 ecotype) grown in jointed medium. Note the bending roots. The concentration of ATP in the lower part is marked on top of the photos. **(B)** Time-lapse images of seedlings grown in jointed medium without (control) or with 0.5 mM ATP (ATP) in the lower part. The photograph time is marked on top of the photos. **(C)** Seedlings grown in U-shaped (left) or diagonal (right) jointed medium containing 0.5 mM ATP. Note the ATP avoidance response of roots. The small white arrows in the figure mark the different bending styles (bent to left or right) of seedlings at different position. **(D)** Seedlings grown in jointed medium containing 0.5 mM of a non-hydrolyzable ATP analog (bz-ATP) or some nucleotide phosphates (ADP, AMP or GTP). In **(B–D)** WS was used as material. Seedlings in **(A,C,D)** were photographed 5 days after transplantation. Triangles in **(A,B,D)** mark the joint line of the two media. In **(C)** the joint line of the two media is marked with a dotted line. The scale bar is showed on right side of **(A)**.

To verify the response of primary roots to eATP, *Arabidopsis thaliana* seedlings were transplanted onto jointed medium containing ATP in the lower part. In the upper part, primary roots grew straight down due to gravitropism. When root tips approached the joint line of the two media, their growth rate markedly decreased and changed orientation such that roots bent or even grew horizontally. The primary roots' growth rate decrement and bending curvature increased with increasing eATP concentration (*p* < 0.05, Student's *t*-test). The root curvature of WS was bigger than of Col-0 (Figure 1A; Figures S1A,B). Lower concentration (≤ 0.1 mM) of ATP did not significantly affect root growth rate and orientation, while higher concentration (≥ 1 mM) of ATP almost ceased root growth, roots were neither elongating nor bending (data not shown).

Time-lapse analysis showed that 1 day after transplantation, the root tip was starting to bend. Over the following 4 days, untreated roots grew rapidly and in a downward direction while roots in 0.5 mM ATP-containing jointed medium grew more slowly and horizontally (Figure 1B; Figures S1C,D). The primary roots' response to ATP was further verified using another U-shape jointed medium. In this medium, a section of untreated 1/2 MS medium was surrounded by 0.5 mM ATP-containing medium on left, lower and right side. Roots of transplanted seedlings bent and grew horizontally to the right side when they approached the upper-to-lower joint line, while the root of the right-most seedling grew horizontally to the left when its tip approached the left-to-right joint line (Figure 1C, left). In a diagonally-jointed medium, roots grew along the joint line (Figure 1C, right). These altered growth orientations gave the appearance that roots were attempting to avoid ATP-containing surroundings.

To verify the specificity of this process, the responses of primary roots to various other nucleotides were investigated. *Arabidopsis* seedlings transplanted onto jointed medium containing 0.5 mM ADP, AMP, or GTP, respectively, displayed primary root bending in response to ADP but not to AMP or GTP (Figure 1D). Root of seedlings grown in ADP grew significantly slower and bent with significantly bigger curvature than untreated seedlings (*p* < 0.05, Student's *t*-test) (Figures S1E,F).

To verify that ATP was acting as a signaling molecule rather than an energy carrier, root growth in response to bz-ATP, a weakly-hydrolysable ATP analog, was investigated. Roots exposed to 0.5 mM bz-ATP showed significantly lower growth rate and higher bending curvature (*p* < 0.05, Student's *t*-test) than control, just similar as the response to ATP (Figure 1D; Figures S1E,F).

### Heterotrimeric G protein α subunit Is involved in root's avoidance response to eATP

**Figure 2:**
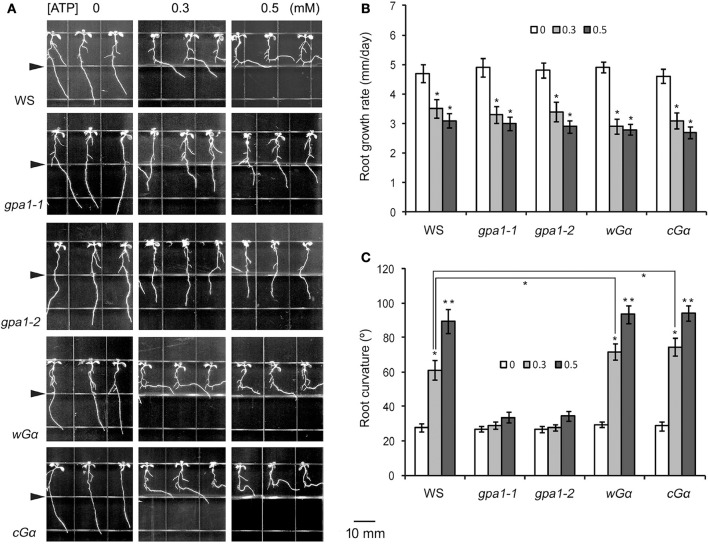
Heterotrimeric Gα is involved in eATP avoidance response. **(A)** Seedlings of WS, Gα null mutants (*gpa1-1, gpa1-2*) and OE lines (*wG*α and *cG*α) grown in jointed medium containing 0, 0.3, and 0.5 mM ATP in the lower part, respectively. Note the different response of various genotypes to ATP. Seedlings were photographed 5 days after transplantation. In each photo series, the triangle marks the joint line of the two media. The scale bar is showed beside. **(B,C)** note the effect of ATP on root growth rate **(B)** and root curvature **(C)**, respectively. In each experiment, root growth rate and curvature of at least 30 seedlings were measured, and data from 3 replicates were calculated to get the mean ± SD. Student's *t*-test *p*-values: ^*^p < 0.05, ^**^p < 0.01.

To analyze the role of heterotrimeric G protein in ATP-affected root growth, the responses of two loss-of-function mutants of the Gα subunit (*gpa1-1, gpa1-2*) to eATP were investigated. In ATP-containing jointed medium, roots of the two mutants did not bend when they approached the joint line. Roots of the two Gα OE lines (*wG*α and *cG*α) bent and grew horizontally when they approached the joint line (Figure 2A).

To analyze the role of Gα in the root response to eATP, root growth rate and curvature were measured. The root growth rates of wild type WS, Gα null mutants and Gα OE lines were all significantly decreased (*p* < 0.05, Student's *t*-test) in ATP-containing jointed medium (Figure 2B), indicating that heterotrimeric Gα is unlikely involved in eATP-suppressed root elongation.

Root curvature measurements showed that in untreated medium, root curvature of WS, Gα null mutants and OE lines was similar. In ATP-containing jointed medium, the degree of root curvature of WS, *wG*α and *cG*α were significantly higher than of the untreated seedlings (*p* < 0.05, Student's *t*-test). However, the degree of root curvature of Gα null mutants (*gpa1-1* and *gpa1-2*) were not significantly different from of untreated seedlings (*p* > 0.05, Student's *t*-test) (Figure 2C). The 0.3 mM ATP induced root curvature increment of *wG*α and *cG*α was significantly bigger than of WS (*p* < 0.05, Student's *t*-test), indicating that the Gα OE lines were more sensitive to ATP than wild type (Figure 2C). These results indicated that the heterotrimeric Gα subunit may play an important role in roots' avoidance response to eATP.

To analyze the role of the heterotrimeric Gβ and Gγ subunit in the roots' response to eATP, the root growth of Gβ null mutants (*agb1-1, agb1-2*) and Gγ null mutants (*agg1-1, agg1-2*) in jointed medium was assessed. Results showed that the null mutants of Gβ or Gγ responded to 0.3, 0.5 mM ATP similarly to the wild type (Col-0), and their primary roots bent and grew horizontally when the root tip approached the joint line (Figure S2A). The growth rate (Figure S2B) and root curvature (Figure S2C) of Gβ or Gγ null mutants were not significantly different to wild type (*p* > 0.05, Student's *t*-test). Therefore, heterotrimeric Gβ and Gγ are unlikely to participate in roots' avoidance response to eATP.

### Heterotrimeric Gα-regulated Ca^2+^ influx is involved in roots' avoidance response to eATP

**Figure 3:**
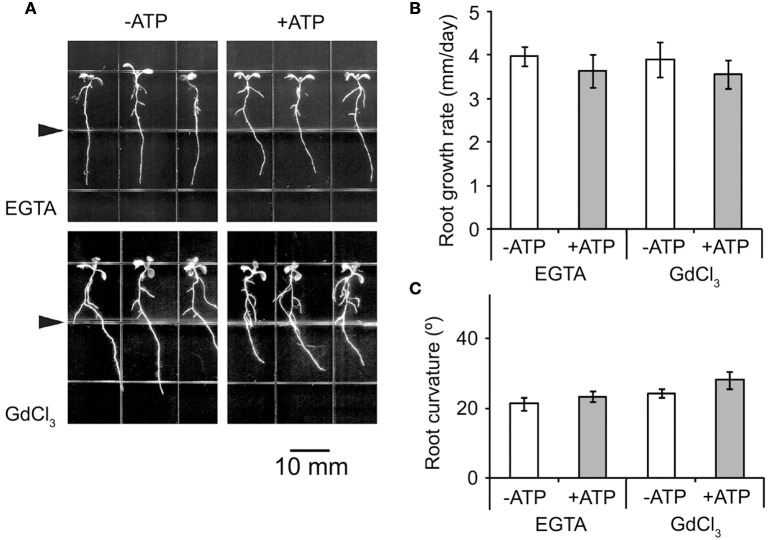
Ca^2+^ influx is necessary for eATP avoidance responses of *Arabidopsis* roots. **(A)** Seedlings of WS grown in jointed medium. EGTA (0.5 mM) or Gd^3+^ (50 μM) were added into 1/2 MS medium, then 0.3 mM ATP was added into the lower part of the jointed medium. The triangle marks the joint line of the two media. The scale bar is showed below. **(B,C)** note the root growth rate **(B)** and root curvature **(C)** before and after ATP treatments. Seedlings were photographed 5 days after transplantation. Primary root growth rate and curvature were measured in at least 30 seedlings, and data from 3 replicates were calculated to get the mean ± SD.

To determine the role of Ca^2+^ in roots' avoidance response to eATP, the effects of the Ca^2+^ chelator EGTA [ethylenebis(oxyethylenenitrilo) tetraacetic acid] and the Ca^2+^ channel blocker GdCl_3_ (Gadolinium tri-chloride) on WS root growth in response to eATP were evaluated. In jointed medium containing 0.5 mM EGTA or 50 μM GdCl_3_ (in both the upper and the lower part), 0.5 mM ATP (in the lower part only) did not significantly suppress root growth rate or lead root growth re-orientation (Figure 3A). The root growth rate and curvature after ATP treatment were not significantly different from control (*p* > 0.05, Student's *t*-test) (Figures 3B,C).

**Figure 4:**
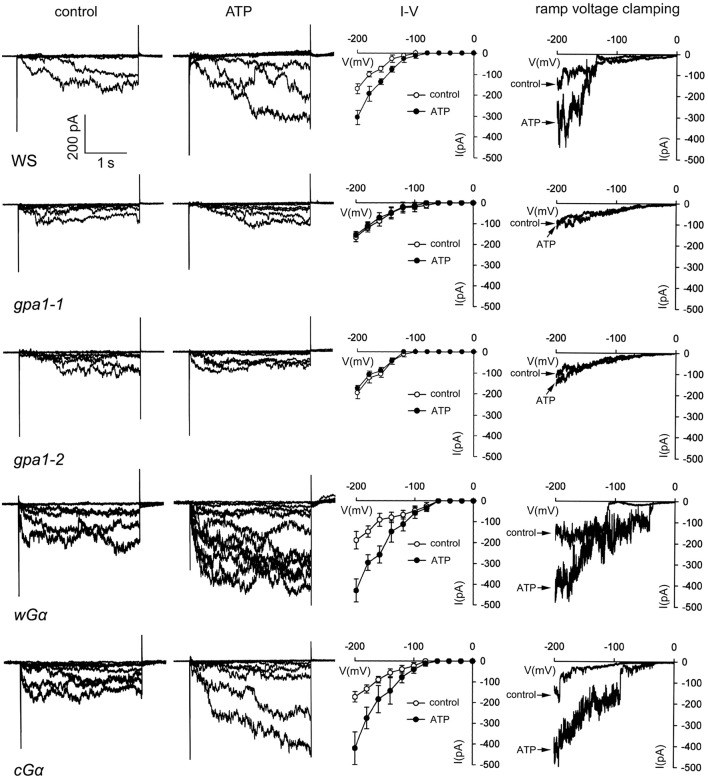
Heterotrimeric Gα is involved in eATP-stimulated Ca^2+^ influx in root cells. Ca^2+^ influx across the PM of root cell protoplasts was detected by whole-cell voltage clamping. Results from step-(1st to 3rd column) and ramp-(4th column) voltage clamping recordings in wild type (WS), Gα null mutants (*gpa1-1* and *gpa1-2*) and OE lines (*cG*α and *wG*α). In each line, current traces before (control), and after 0.1 mM ATP treatment are shown in the 1st and 2nd column, respectively. The 3rd column shows the I–V relationship curve of step-voltage clamping (*n* = 6). The 4th column notes the current traces before and after 0.1 mM ATP treatment which were recorded by slow ramp-voltage clamping. The genotype is marked below each line on the left. The time/current intensity scale bar is showed on the lower left of the first line.

To further confirm the involvement of Ca^2+^ influx in eATP regulated growth response, whole-cell voltage patch clamping was used to detect Ca^2+^ influx across the PM of root cells. In protoplasts of WS root cells, a hyperpolarization-activated Ca^2+^ influx current was recorded. The channel opened around a membrane voltage of −100 mV, and current intensity increased with increasing membrane hyperpolarization. After 0.1 mM ATP stimulation, max Ca^2+^ influx current intensity was significantly stronger than in the control (*p* < 0.05, Student's *t*-test) (Figure 4).

To analyze the role of heterotrimeric Gα in ATP-stimulated Ca^2+^ influx, the effect of ATP on Ca^2+^ influx in root protoplasts of Gα null mutants and OE lines was investigated. Results showed that 0.1 mM ATP only slightly affected Ca^2+^ influx in *gpa1-1* and *gpa1-2*, and the Ca^2+^ influx current intensity did not significantly increase (*p* > 0.05, Student's *t*-test). In contrast, 0.1 mM ATP remarkably stimulated Ca^2+^ influx into root protoplasts of the two Gα OE lines. The max current intensity at −200 mV significantly increased (*p* < 0.05, Student's *t*-test). ATP-stimulated Ca^2+^ current intensity increment in Gα OE lines were significantly higher than that in WS (*p* < 0.05, Student's *t*-test). Furthermore, the ramp voltage clamping results, which showed that ATP remarkably stimulated Ca^2+^ influx in WS and Gα OE lines but not in Gα null mutants, confirming the involvement of Gα in ATP-stimulated Ca^2+^ influx (Figure 4).

### Heterotrimeric Gα is involved in eATP regulated asymmetric auxin response reporter activity in root tip cells

**Figure 5:**
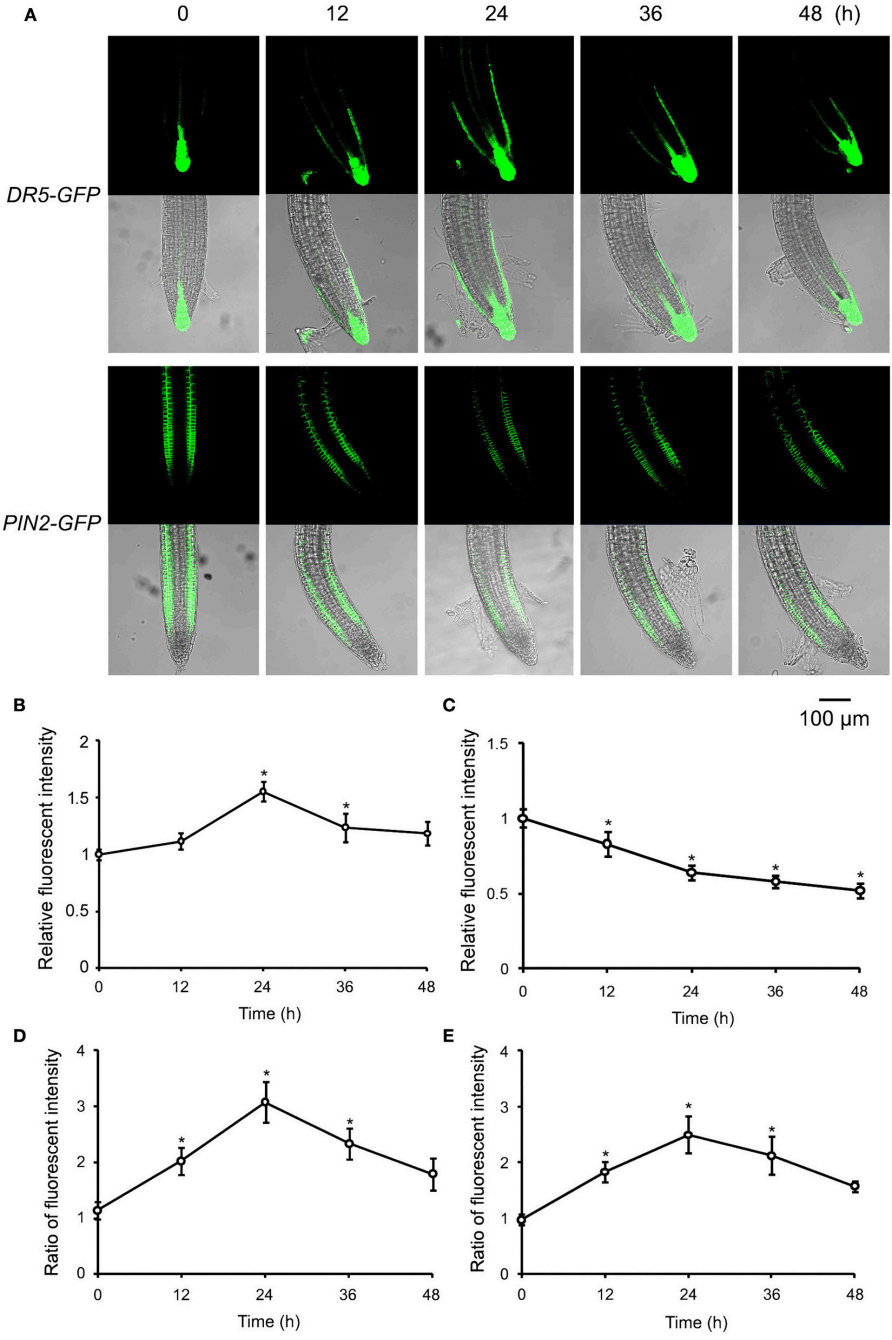
ATP-induced asymmetric distribution of DR5-GFP and PIN2-GFP in root tip cells. *DR5-GFP* or *PIN2-GFP* transgenic WS was used as material. Seedlings were grown in 1/2 MS medium for 4 days and transplanted onto 0.5 mM ATP-containing jointed medium. After 12, 24, 36, and 48 h, the fluorescence of DR5-GFP or PIN2-GFP in root tip cells was detected using confocal laser scanning microscopy (CLSM). **(A)** Fluorescence in representative root tips. In each series, the upper and lower line note the images of fluorescence and merged fluorescence/transmission, respectively. The scale bar is showed below. **(B,C)** note time-lapse analysis result of relative fluorescence intensity (the ratio of total fluorescence intensity after/before ATP treatment) of DR5-GFP **(B)** or PIN2-GFP **(C)** in root tip cells, respectively. **(D,E)** Note time-lapse analysis results of the ratio of fluorescence intensity (fluorescence intensity in right-side cells relative to that in left-side cells) of DR5-GFP **(D)** and PIN2-GFP **(E)**, respectively. In each experiment, fluorescence intensity in up to 10 roots was measured. Data from 3 replicates were calculated to get the mean ± SD. Student's *t*-test *p*-values: ^*^p < 0.05.

Auxin plays a key role in the tropism of plant organs. To verify the possible role of auxin in roots' eATP avoidance response, the auxin response reporter (DR5-GFP) activity in root tip cells was detected. In untreated WS roots, DR5-GFP fluorescence was mainly located around the quiescent center in root tips. eATP led to significant increase of the fluorescence intensity (*p* < 0.05, Student's *t*-test) in quiescent center and epidermal cells of meristem and transition zone (Figures 5A,B). Twelve hours after transplantation, asymmetric distribution of fluorescence appeared in the elongation zone, fluorescence intensity in the inner side of the root curve was significantly higher than in the outer side (*p* < 0.05, Student's *t*-test) (Figures 5A,D), indicating that the auxin response reporter activity may be higher in cells in the inner side. This asymmetric distribution persisted at 24 and 36 h after transplantation. At 48 h, the fluorescence intensity in root tip cells became weaker (Figures 5A,D).

To determine the underlying mechanism responsible for the asymmetric distribution of auxin response reporter activity, the abundance and location of the auxin transporters were evaluated in ATP-treated root cells. Since PIN2 has been reported to be located mainly in epidermal cells of meristem and transition zone and to play a key role in root tropism (Sun et al., 2008; Lin et al., 2012), its abundance and distribution were assessed first. In the untreated root tip, PIN2-GFP fluorescence was located in epidermal cells of meristem and transition zone while not in the quiescent center and surrounding cells. 12 h after 0.5 mM ATP treatment, the fluorescence intensity of PIN2-GFP decreased dramatically (Figures 5A,C). Fluorescence intensity in the inner side of root curve was significantly higher than in the outer side (*p* < 0.05, Student's *t*-test), suggesting that PIN2 may be more abundant in the inner-side cells. Such an asymmetric distribution stably persisted in root tips 24 h and 36 h after treatment and disappeared 48 h after treatment (Figures 5A,E).

To further investigate the effect of eATP on the content and distribution of other auxin transporters, the fluorescence intensity and signal distribution in root tip cells of *PIN1-GFP, PIN3-GFP*, or *PIN7-GFP* transgenic plants was analyzed. PIN1-GFP fluorescence was mainly located in the developing vessel cells, PIN3-GFP and PIN7-GFP fluorescence was mainly located in root cap cells and developing vessel cells. 24 h after 0.5 mM ATP treatment, the fluorescence intensity of PIN1-GFP, PIN3-GFP, and PIN7-GFP decreased markedly while their location did not change (Figure S3).

**Figure 6:**
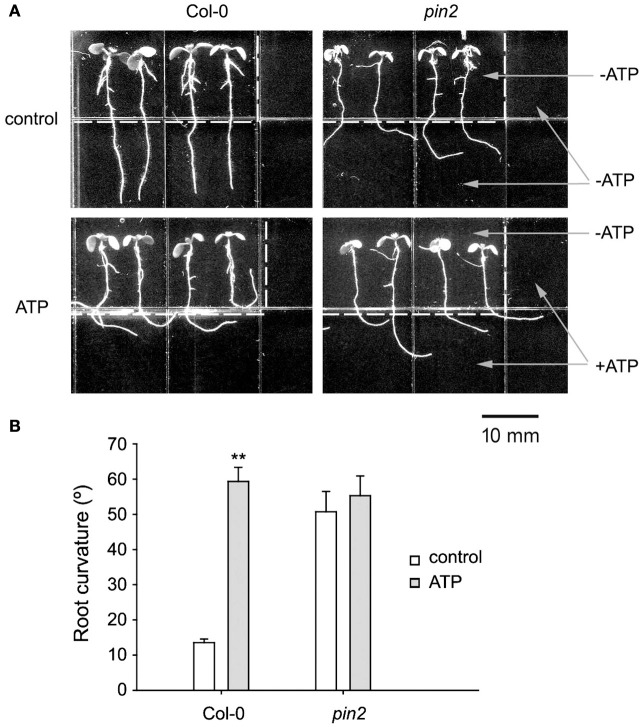
PIN2 null mutant did not respond to eATP. **(A)** Seedlings of Col-0 and PIN2 null mutant (*pin2*) grown in U-shape jointed medium containing 0 (control) or 0.5 mM ATP (ATP) in the lower part and two sides, respectively. Seedlings were photographed 5 days after transplantation. The dotted line marks the joint line of the two media. The scale bar is showed below. **(B)** Effect of ATP on root curvature. Root curvature of at least 30 seedlings was measured, and data from 3 replicates were calculated to get the mean ± SD. Student's *t*-test *p*-values: ^**^p < 0.01.

To confirm the role of PIN2 in roots' avoidance response to eATP, response of primary roots of *pin2* was investigated. In untreated medium, root growth orientation of *pin2* seedlings was randomized, showing the impaired gravitropism. In U-shaped jointed medium which contained 0.5 mM ATP, roots of Col-0 bent and grew horizontally, root of the rightmost seedling bent or even grew upward along the joint line; while root growth orientation of *pin2* was similar to untreated seedlings (Figure 6A). Data analysis showed that ATP significantly induced root growth re-orientation of Col-0 seedlings (*p* < 0.01, Student's *t*-test); however, root curvature of *pin2* seedlings did not significantly change (*p* > 0.05, Student's *t*-test) (Figure 6B). These results confirmed that PIN2 may be necessary for roots' avoidance response to eATP.

**Figure 7:**
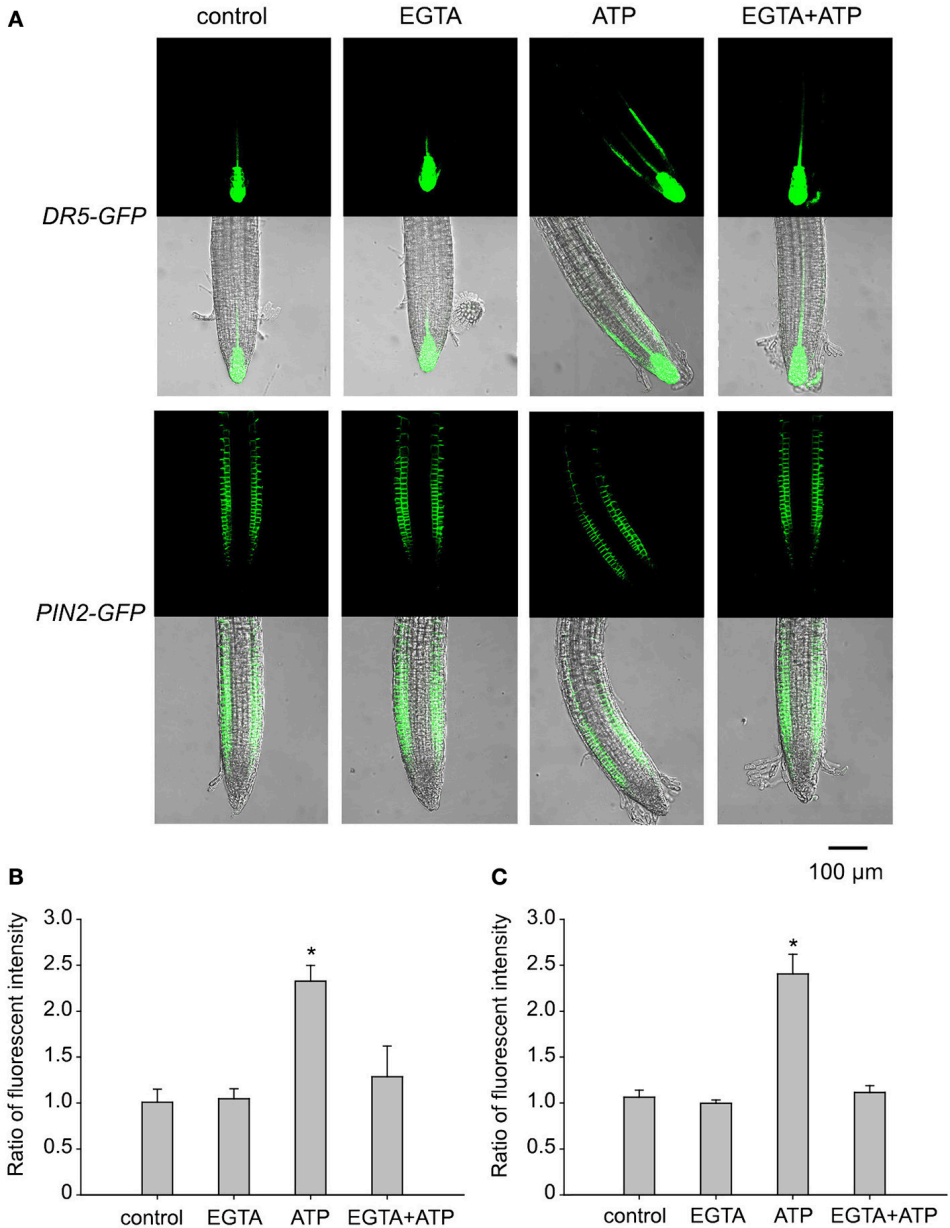
Ca^2+^ is involved in eATP-induced asymmetric distribution of DR5-GFP and PIN2-GFP. *DR5-GFP* or *PIN2-GFP* transgenic WS seedlings grown in 1/2 MS medium for 4 days were transplanted onto jointed medium containing 0.5 mM EGTA (in both the upper and the lower part) and 0.5 mM ATP (in the lower part only). After 24 h, fluorescence was detected by using CLSM. **(A)** Fluorescence in representative root tip cells. The scale bar is showed below. **(B,C)** note the fluorescence intensity ratio (fluorescence intensity in right-side cells relative to that in left-side cells) of DR5-GFP **(B)** and PIN2-GFP **(C)**, respectively. In each experiment, fluorescence intensity in up to 10 roots was measured. Data from 3 replicates were calculated to get the mean ± SD. Student's *t*-test *p*-values: ^*^p < 0.05.

To assess the role of Ca^2+^ in ATP-induced asymmetric distribution of DR5-GFP and PIN2, the effect of EGTA on their fluorescence intensity and distribution was investigated. In EGTA containing jointed medium, fluorescence intensity and distribution of DR5-GFP or PIN2-GFP did not change significantly after 0.5 mM ATP treatment (Figure 7A). The ratio of DR5-GFP or PIN2-GFP fluorescence intensity in right-side cells relative to in left-side cells was not significantly affected by ATP (*p* > 0.05, Student's *t*-test) (Figures 7B,C).

**Figure 8:**
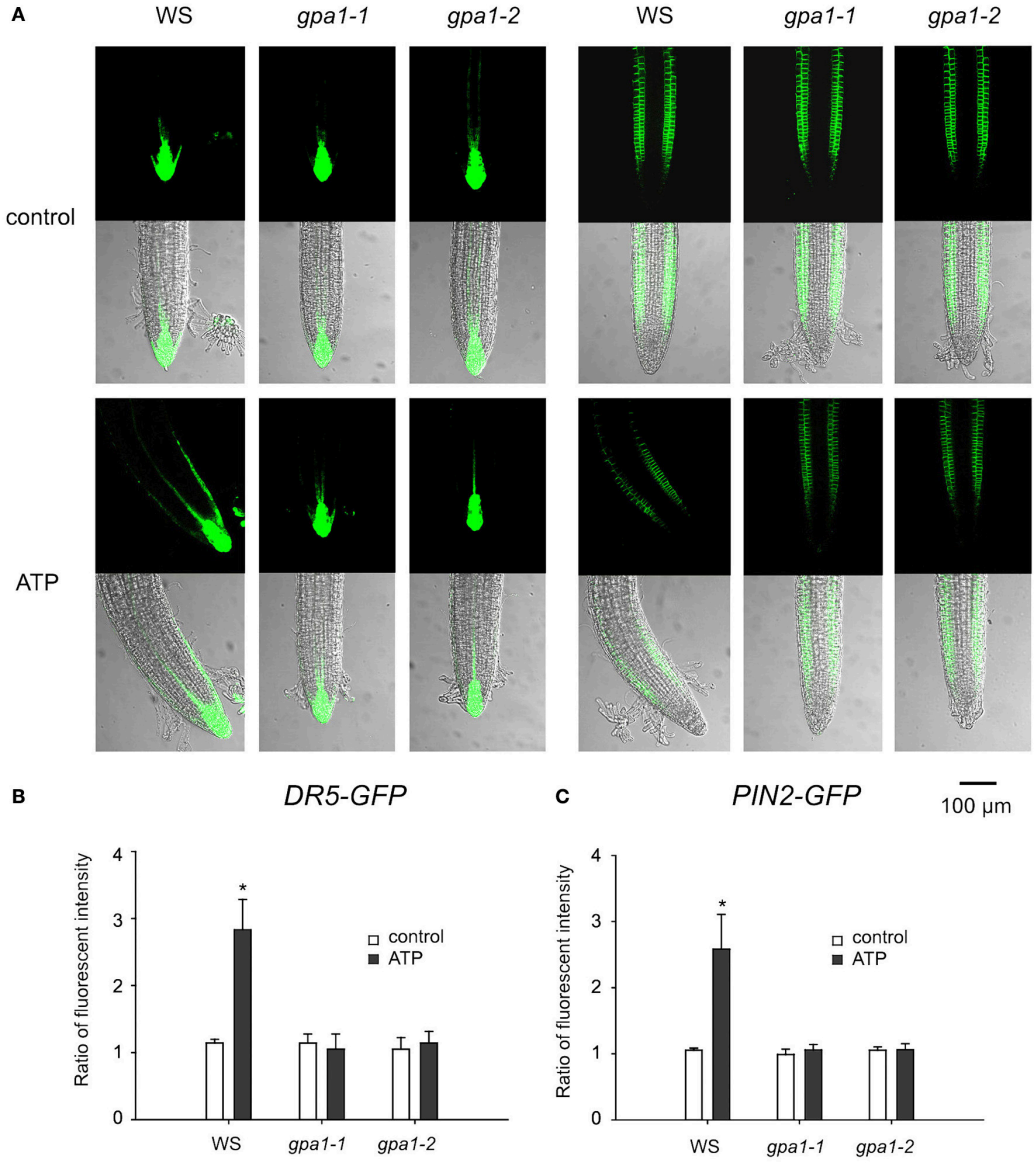
Heterotrimeric Gα is involved in eATP-induced asymmetric distribution of DR5-GFP and PIN2-GFP. *DR5-GFP* or *PIN2-GFP* transgenic WS, *gpa1-1* and *gpa1-2* seedlings were grown in 1/2 MS medium for 4 days and then transplanted onto 0.5 mM ATP-containing jointed medium. After 24 h, fluorescence was detected by using CLSM. **(A)** Fluorescence in representative root tip cells. The scale bar is showed below. **(B,C)** note fluorescence intensity ratio (fluorescence intensity in right-side cells relative to that in left-side cells) of DR5-GFP **(B)** and PIN2-GFP **(C)**, respectively. In each experiment, fluorescence intensity in up to 10 roots was measured. Data from 3 replicates were calculated to get the mean ± SD. Student's *t*-test *p*-values: ^*^p < 0.05.

To verify the role of heterotrimeric Gα in ATP-induced asymmetric distribution of DR5-GFP and PIN2-GFP, Gα null mutants were transformed with *DR5-GFP* or *PIN2-GFP* and used to detect the fluorescence in root tip cells. In untreated seedlings, the fluorescence intensity and distribution of DR5-GFP and PIN2-GFP were similar between wild type and the two Gα null mutants. After 0.5 mM ATP treatment, fluorescence intensity of DR5-GFP in root tip cells of Gα null mutants did not change, and the fluorescence intensity of PIN2-GFP decreased. However, neither DR5-GFP nor PIN2-GFP was found to be asymmetrically distributed in root tip cells of the two Gα null mutants (Figure 8A). In *gpa1-1* and *gpa1-2*, ATP did not significantly affect the ratio of DR5-GFP or PIN2-GFP fluorescence intensity in right-side cells relative to in left-side cells at the root tip (*p* > 0.05, Student's *t*-test) (Figures 8B,C).

To further confirm the role of Gα in roots' eATP avoidance response, seedlings of WS and Gα null mutants were transplanted onto diagonally-jointed medium which containing ATP on right-lower side. When root tip approached the jointed line, roots of WS bent to left, however, roots of *gpa1-1* and *gpa1-2* grew straightly downward (Figure S4A). In root tip of WS seedlings, after such an asymmetric ATP stimulation, fluorescence intensity of DR5-GFP or PIN2-GFP in left-side cells was higher than in right-side cells (Figure S4B). And the ratio of DR5-GFP or PIN2-GFP fluorescence intensity in left-side cells relative to right-side cells was significantly higher than that in untreated seedlings (*p* < 0.05, Student's *t*-test). However, the fluorescence intensity ratio did not significantly change in root tip cells of *gpa1-1* and *gpa1-2* (*p* > 0.05, Student's *t*-test) (Figures S4C,D).

### Heterotrimeric Gα is involved in eATP-regulated functional gene expression in root cells

**Figure 9:**
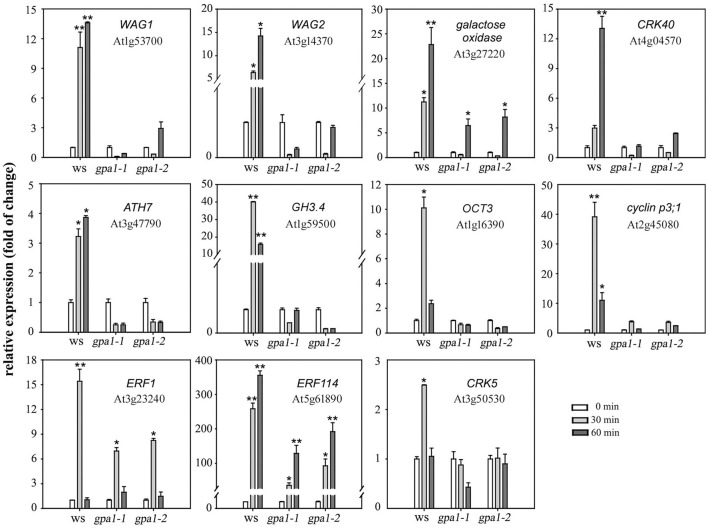
Heterotrimeric Gα is involved in eATP-regulated functional gene expression. Seedlings grown in 1/2 MS medium for 4 days were transplanted onto a 0.5 mM ATP-containing jointed medium. After 30 and 60 min, roots were cut to extract total RNA, and the expression levels of several functional genes were measured by real-time qPCR. The relative level (fold of change) of gene expression in WS and Gα null mutants (*gpa1-1* and *gpa1-2*) is showed. Gene names and the corresponding gene loci are noted in each figure. Data from 3 replicates were calculated to get mean ± SD. Student's *t*-test *p*-values: ^*^p < 0.05, ^**^p < 0.01.

To further clarify the role of heterotrimeric Gα in eATP avoidance response, the gene expression pattern in root tip cells was detected by DNA microarray and real-time qPCR analysis. Results of DNA microarray showed that, 60 min after 0.5 mM ATP treatment, 650 genes were up-regulated and 765 genes were down-regulated in WS, 622 genes were up-regulated and 702 genes were down-regulated in *gpa1-2* (Figure S5). Expression of genes, which were significantly up-regulated in WS while not up-regulated in Gα null mutants, was detected by using realtime q-PCR. The transcriptional response to ATP of several genes differed significantly between wild type and Gα null mutants. The expression of 11 functional genes, including *WAG1* and *WAG2, CRK40, cyclin p3;1, ERF1, ERF114*, and *CRK5*, was significantly enhanced by 0.3 mM ATP in WS plants (*p* < 0.05, Student's *t*-test) while in *gpa1-1* and *gpa1-2*, their expression either did not significantly increase (e.g., *WAG1, WAG2, CRK40, ATH7, GH4, OCT3, cyclin p3;1*, and *CRK5*; *p* > 0.05, Student's *t*-test) or was less effectively promoted (e.g., galactose oxidase gene, *ERF1*, and *ERF114*; Figure 9) (The full name and function of these genes encoding proteins are explained in Table S2 in detail). These results indicated that heterotrimeric Gα may be involved in ATP-regulated expression of functional genes.

### DORN1 is unlikely involved in eATP avoidance response of *arabidopsis* roots

To analyze the role of the P2K receptor (DORN1) in the roots' response to eATP, the root growth of DORN1 null mutants (*dorn1-1, dorn1-3*) in jointed medium was assessed. The two mutants responded to 0.3, 0.5 mM ATP similarly to the wild type (Col-0), primary roots bent when they approached the joint line (Figure S6A). The growth rate and root curvature of Col-0 and DORN1 null mutants were all significantly affected by ATP (*p* < 0.05, Student's *t*-test) (Figures S6B,C). The ATP induced decrement of root growth rate and increment of root curvature were not significantly different between Col-0 and the two mutants (*p* > 0.05, Student's *t*-test), indicating that DORN1 is unlikely to participate in roots' avoidance response to eATP.

## Discussion

Extracellular ATP (eATP) regulates root growth and leads to a reduced root growth rate and a bending growth phenotype in *Arabidopsis* roots (Tang et al., 2003; Liu et al., 2012). Root skewing on surface of tilted solid medium was also promoted by added ATP (Yang et al., 2015). Nevertheless, the nature and physiological significance of eATP-regulated root growth is still unclear. Here, in a transitional circumstance, primary roots grow gradually from ATP-free to ATP-containing conditions, making the clear detection of ATP sensing and response processes possible. A similar method had previously been used to investigate the root response to NaCl (Sun et al., 2008).

When the root tip approached the joint line of the two media, the reduction in growth rate and re-orientation of roots indicated that the root tip sensitively sensed eATP and attempted to avoid ATP-containing conditions. It appeared as though roots recognize high concentrations of ATP as harmful “danger signals.” It has previously been reported that ATP may act as a “damage-associated molecular pattern” (DAMP) to plant cells (Choi et al., 2014b). Here, by using jointed medium, roots' avoidance response to high concentration of ATP was clearly showed. Based on results in this work, we speculate that growth re-orientation may be a response style that helps roots recognize and escape from danger.

Tang et al. (2003) reported that *Arabidopsis* root growth in ATP media showed wavy or skewing phenotypes, which looked different from the bending phenotype here. In this work, jointed medium gave an asymmetric stimulation, that may be the reason for the difference of roots' phenotype. Tang et al. (2003) and Liu et al. (2012) used 1.5 and 1.2% agarose in plant growth media, respectively. We have compared seedlings grown in 0.8 and 1.0% phytagel containing medium. The roots' response to eATP was similar, so we believe the content of phytagel did not affect phenotype. eATP induced root bending or curling had been regarded as disrupted gravitropic response (Tang et al., 2003; Liu et al., 2012). However, in this work, roots' gravitropic response was unlikely disrupted, e.g., in diagonal jointed medium, roots' gravitropism clearly existed. The reported ATP-induced root circling (Tang et al., 2003) and ATP-promoted root skewing (Yang et al., 2015) might be the response to uniform ATP conditions, i.e., the roots perceived ATP and tried to escape, but the continuous escaping eventually resulted in root curling or strengthened waving. This result elucidated the physiological relevance of eATP-induced root bending and expanded our knowledge of eATP's physiological function as a danger signal.

Auxin plays essential roles in plant cell proliferation and elongation and participates in the growth and development of plant organs. In roots, intracellular auxin levels were subtly regulated by a complicated transport system containing both auxin efflux and influx transporters. In the root tip, several PIN-FORMED proteins are located in different parts and control auxin transport between the tip and the base (Band et al., 2014). Auxin levels are normally low in root cells, increased auxin level inhibit root cell elongation (Barbez et al., 2017). Auxin accumulation in root cells stimulated by eATP has been reported and is thought to be the reason for ATP-inhibited root elongation (Tang et al., 2003; Liu et al., 2012; Yang et al., 2015). Auxin levels in root tip cells elevated markedly when the root tip comes into contact with ATP, especially in the quiescent center, the dividing and elongating area (Figure 5). Such an eATP-induced auxin accumulation may possibly result in suppressed root cell elongation.

Plant growth has remarkable plasticity in order to rapidly respond to the continuously changing environment. Plant organ growth responds tropically or negative-tropically to environmental stimuli to capture beneficial elements or avoid harmful elements in their surroundings. Auxin is involved in tropism or negative tropism of plant organs to various stimuli, e.g., the gravitropism of roots and or the negative gravitropism of shoots. Asymmetric distribution of auxin in plant organs is a typical response to unidirectional stimuli (Takahashi et al., 2009; Galvan-Ampudia and Testerink, 2011; Zadnikova et al., 2015). Here, the asymmetric distribution appeared in root tip cells after they sensed eATP, and auxin concentrations were higher in the inner side of the bending area than in the outer side. Since root cells are very sensitive to auxin, the high concentration of auxin may led to inhibition of cell elongation, and the unbalanced elongation rate of root tip cells eventually led to root bending. eATP has been reported to be involved in auxin accumulation in root and hypocotyl cells resulting in suppressed organ elongation and ceased gravitropism (Liu et al., 2012). Liu et al. (2012) reported that high concentration (0.8 mM) ATP diminished gravity-induced asymmetric distribution of DR5-GFP. In contrast, ATP induced asymmetric distribution of DR5-GFP in this work. It looks that, unlike uniformed stimulation, unidirectional ATP stimulation may lead to asymmetric distribution of auxin and root growth re-orientation.

Auxin transporters in the PM are involved in asymmetric distribution of auxin (Laskowski et al., 2008; Band et al., 2014; Sato et al., 2015), and asymmetric distribution of PIN proteins thus plays an essential role in root tropic and avoidance growth (Rahman et al., 2010; Lin et al., 2012; Zhang et al., 2013). In the root tip, PIN2 is located mainly in the transition zone and plays key roles in auxin transport from the root tip to the base (Band et al., 2014). The asymmetric distribution of auxin in root tip cells may result from asymmetrically distributed PIN2 (Sun et al., 2008; Lin et al., 2012; Liu et al., 2012). Here, the asymmetric distribution of PIN2 in root tip cells was identical to the observed asymmetric distribution of auxin response reporter activity, both of which were higher in the inner side cell than in the outer side cell at the root curve. Together with the result that PIN2 null mutant did not respond to ATP stimulation, we suggest that the asymmetric distribution of PIN2 may possibly lead to unbalanced auxin transport which then result in the asymmetric distribution of auxin.

It was previously discovered that eATP stimulate transient increase or oscillation of cytosolic Ca^2+^ concentrations (Demidchik et al., 2009; Tanaka et al., 2010) mediated by Ca^2+^ influx through PM Ca^2+^ channels (Demidchik et al., 2003, 2009; Jeter et al., 2004; Hao et al., 2012; Wang et al., 2014). Nevertheless, the physiological relevance of eATP induced Ca^2+^ influx is not clear. Here, we provide more evidence to verify that Ca^2+^ influx via hyperpolarization-activated Ca^2+^ channel may participate in eATP avoidance response as an important secondary messenger. Ca^2+^ signaling is involved in auxin dynamics in plant cells (Rigo et al., 2013; Li et al., 2016). In this work, the EGTA-suppression on eATP-induced auxin and PIN2 asymmetric distribution in root tip cells further indicated that Ca^2+^ influx may be required for ATP-regulated auxin transport and accumulation.

eATP-regulated gene expression was preliminarily investigated using DNA microarray analysis or proteomic methods (Chivasa et al., 2011; Choi et al., 2014a; Lim et al., 2014). Here, eATP up- and down-regulated expression of hundreds of genes. Most of these genes were up- or down-regulated both in wild type and Gα null mutants. The eATP-induced expression pattern of most genes in this work were identical to the reported results (Chivasa et al., 2011; Choi et al., 2014a; Lim et al., 2014). However, when we analyze gene expression differences between wild type and Gα null mutants, only several functional genes were revealed to be differently expressed in WS and Gα null mutants. These genes encoded proteins that have been reported to be involved in auxin transport and root growth (see detailed information in Table S2). It appears likely that these genes are involved in regulating auxin levels in root cells or other downstream cellular events which will lead either directly or indirectly to roots' avoidance response to ATP. Expression and possible function of these Gα-regulated genes were not focused on in the reported works (Chivasa et al., 2011; Choi et al., 2014a; Lim et al., 2014). The detection of these genes provided new clues to verify the mechanism of roots' response to eATP.

Heterotrimeric G protein plays multiple roles in plant growth and development. Loss-of-function mutants of heterotrimeric G protein displayed altered shoot meristem growth (Bommert et al., 2013), zygote division (Yu et al., 2016), seedling development (Chen et al., 2006; Booker et al., 2010; Jaffe et al., 2012), root architecture (Mudgil et al., 2016) and resistance to pathogens (Llorente et al., 2005; Trusov et al., 2006; Liu et al., 2013). In eATP-induced responses, heterotrimeric G protein participated in stomatal movement (Hao et al., 2012) and obstacle avoidance of roots (Weerasinghe et al., 2009). Here, heterotrimeric Gα was further shown to be involved in the root response to eATP and the subsequent bending growth.

Ion channels in the PM play important roles in the heterotrimeric G protein- triggered signaling cascade (Wang et al., 2001; Wu, Y. et al., 2007; Fan et al., 2008; Zhang et al., 2011). Here, Ca^2+^ channel in PM of root cells was revealed to be regulated by heterotrimeric G protein, together with the observation that eATP-induced root bending was blocked by EGTA and Gd^3+^, supporting the role of Ca^2+^ influx in this process. We thus suggest that heterotrimeric G protein-regulated Ca^2+^ influx may be a fundamental signaling event in the roots' response to eATP.

As mentioned above, asymmetric auxin distribution is a marked response in the bending growth of roots. Heterotrimeric G protein has been reported to be involved in auxin transport by regulating the expression and distribution of auxin transporters in root cells (Pandey et al., 2008; Mudgil et al., 2009; Booker et al., 2010). Here, we provided additional data to show that heterotrimeric Gα may possibly be involved in the eATP-regulated activity and distribution of PIN2 which ultimately led to the asymmetric accumulation of auxin.

Combining these results, we speculate that heterotrimeric Gα participates in the eATP-mediated avoidance growth of roots by stimulating Ca^2+^ channels, and the subsequent Ca^2+^ influx alters the activity and distribution of PIN2, leading to the asymmetric distribution of auxin, and the expression of functional genes related to cell proliferation and elongation, leading to asymmetric elongation and eventually root bending growth. It has been reported that heterotrimeric G protein is involved in eATP sensing and response (Weerasinghe et al., 2009; Hao et al., 2012). Nevertheless, the heterotrimeric G protein regulated cellular events were not clearly discovered. The results here further elucidating the role of heterotrimeric G protein in roots' response to eATP.

Although heterotrimeric G protein is located in the PM of plant cells, it is not able to bind any ligand and get directly activated. In the PM of plant cells, many receptor-like kinases had been reported to be involved in growth, development and stress responses. Recent evidence showed that some receptor-like kinases play essential roles in coupling various stimuli with the activation of heterotrimeric G proteins (Bommert et al., 2013; Aranda-Sicilia et al., 2015; Yu et al., 2016). The first reported eATP receptor in plant cell, a lectin receptor kinase, DORN1, has been shown to participate in several eATP-induced responses (Choi et al., 2014a). However, result in this work indicated that DORN1 is unlikely involved in roots' avoidance response to eATP. The reason need to be further revealed. The possibility that some receptor-like kinases, which can interact with heterotrimeric G protein, may function as eATP sensors needs to be further verified.

## Author contributions

RZ performed gene transformation, auxin; PIN detection and provided assistance to ZS; XD performed cross hybridization and qPCR experiments and provided assistance to ZS and TL; WH performed root growth parameter measurement; WG and WZ performed patch clamp experiments; SX provided technical assistance to WH; TL designed the experiments and analyzed the data; ZS conceived the research plans, supervised and complemented the writing.

### Conflict of interest statement

The authors declare that the research was conducted in the absence of any commercial or financial relationships that could be construed as a potential conflict of interest.
