# Supplementary material for: Heterotrimeric G Protein-Regulated Ca2+ Influx and PIN2 Asymmetric Distribution Are Involved in Arabidopsis thaliana Roots' Avoidance Response to Extracellular ATP
Source: Front Plant Sci. 2017 Sep 1;8:1522. doi: 10.3389/fpls.2017.01522 (PMC5585194; doi:10.3389/fpls.2017.01522)
Supplement: Supplementary file 1 [file Presentation1.pdf]

SUPPLEMENTARY FILES

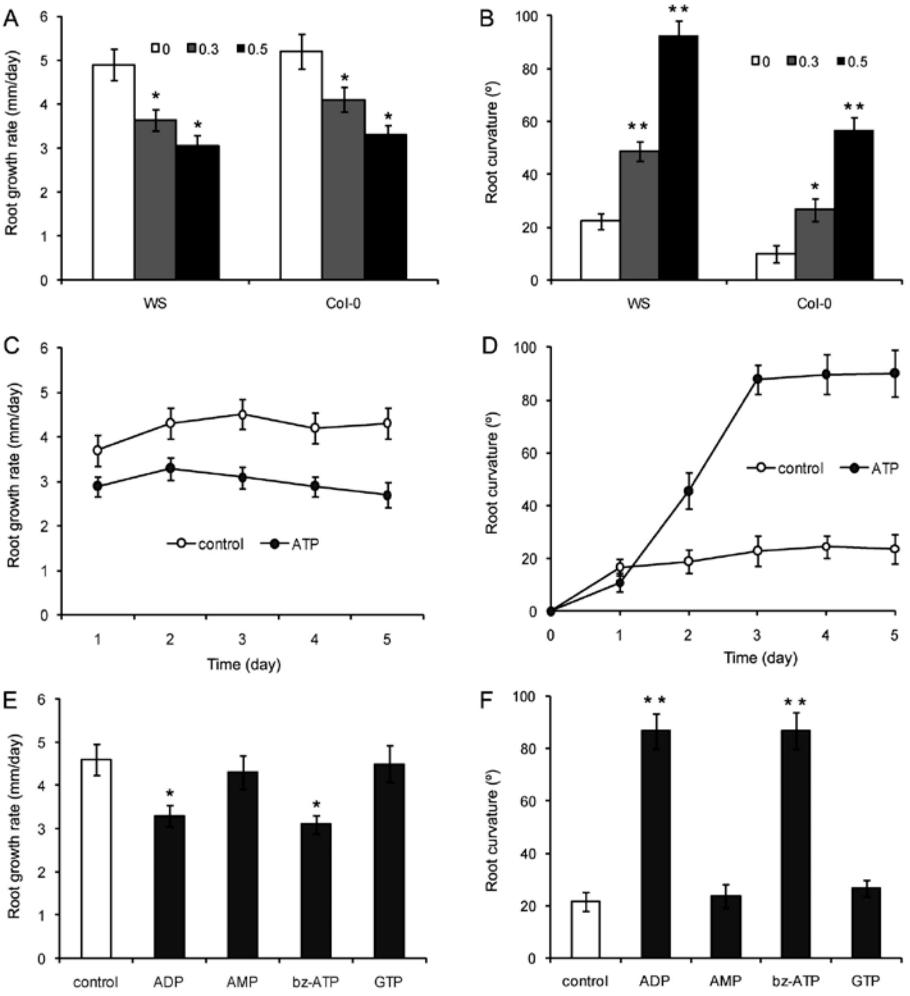

FIGURE S1│ Data analysis of eATP avoidance response of *Arabidopsis* roots. **(A,B)** Root growth rate **(A)** and root curvature **(B)** of *Arabidopsis thaliana* (WS and Col-0 ecotype) seedlings grown in jointed medium for 5 days, note the dose-dependence of ATP induced response. **(C,D)** Show time-lapse analysis of root growth rate **(C)** and root curvature **(D)** of seedlings grown in 0.5 mM ATP-containing jointed medium, respectively. **(E,F)** Show root growth rate **(E)** and root curvature **(F)** of seedlings grown for 5 days in jointed medium containing 0.5 mM of bz-ATP, ADP, AMP or GTP, respectively. In **(C-F)**, WS was used as material. In each experiment, at least 30 seedlings were measured. Data from 3 replicates were calculated to get the mean ± SD. Student's *t*-test *p*-values: * $p<0.05$, ** $p<0.01$.

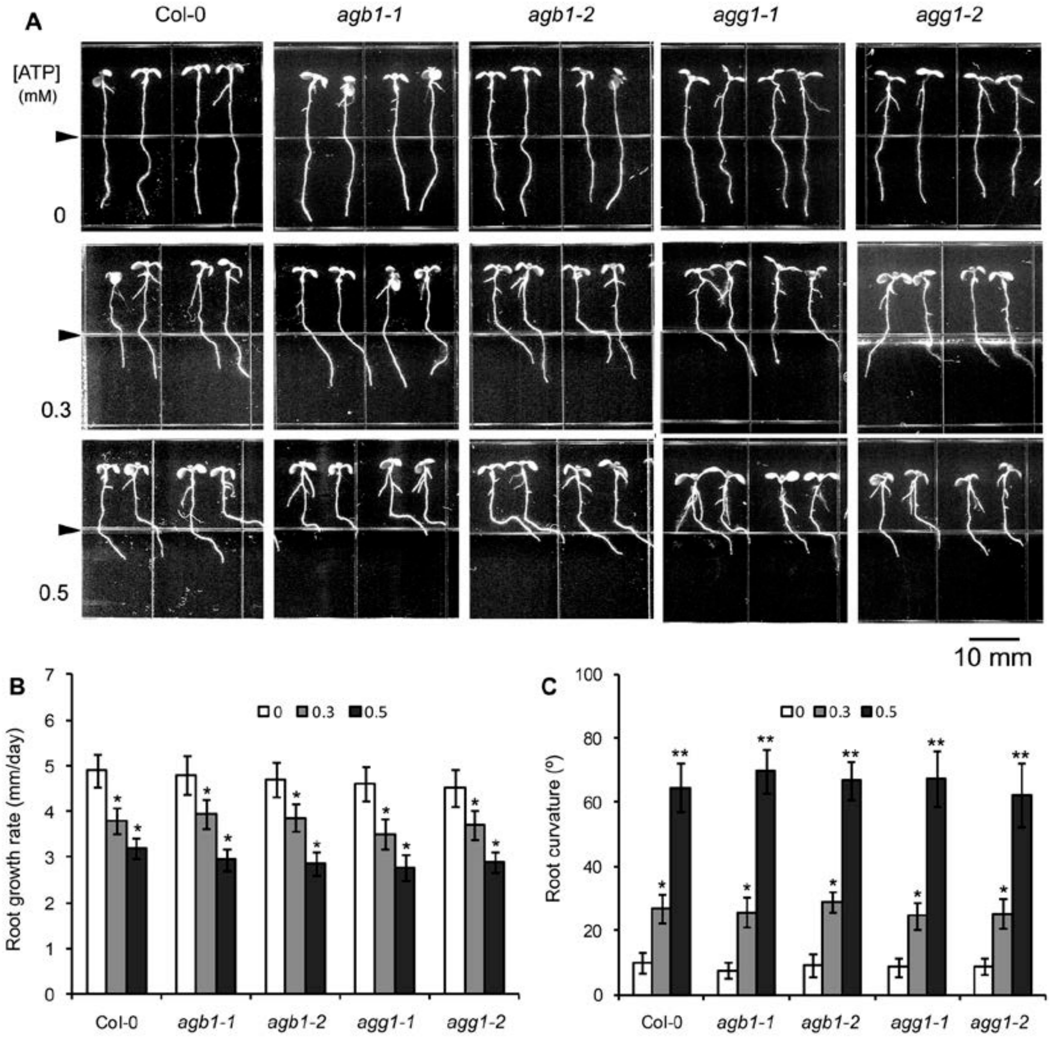

FIGURE S2│Heterotrimeric Gβ and Gγ are unlikely involved in eATP avoidance response of *Arabidopsis* roots. **(A)** Seedlings of Col-0, Gβ null mutants (*agb1-1*, *agb1-2*) and Gγ null mutants (*agg1-1, agg1-2*) grown in jointed medium containing 0, 0.3 and 0.5 mM ATP in the lower part, note the response of various genotypes to ATP. Seedlings were photographed 5 days after transplantation. In each photo series, the triangle marks the joint line of the two media. The scale bar is showed below. **(B,C)** Note the root growth rate **(B)** and root curvature **(C)**, respectively. In each experiment, at least 30 seedlings were measured. Data from 3 replicates were calculated to get the mean ± SD. Student's *t*-test *p*-values: * $p<0.05$, ** $p<0.01$.

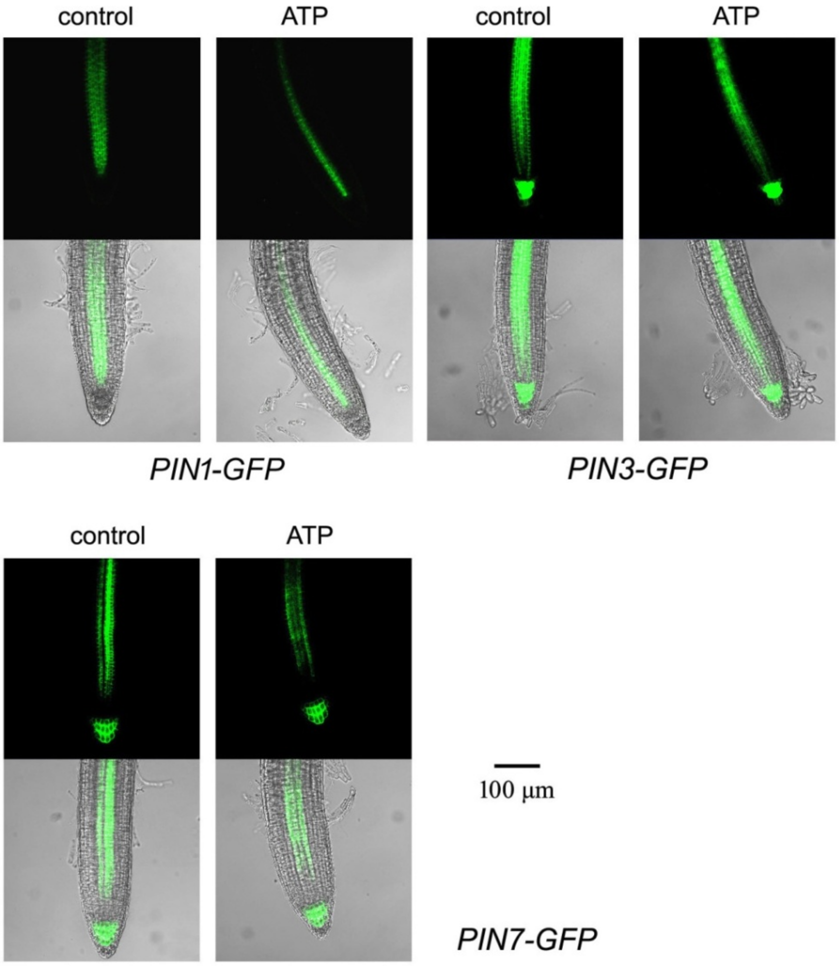

FIGURE S3│The effect of eATP on abundance and distribution of PIN1, PIN3 and PIN7 in root tip cells. *PIN1-GFP*, *PIN3-GFP* or *PIN7-GFP* transgenic wild type (Col-0) seedlings were grown in 1/2 MS medium for 4 days and then transplanted onto 0.5 mM ATP-containing jointed medium. After 24 h, fluorescence in root tip cells was detected using CLSM. Scale bar is showed beside the image of PIN7-GFP.

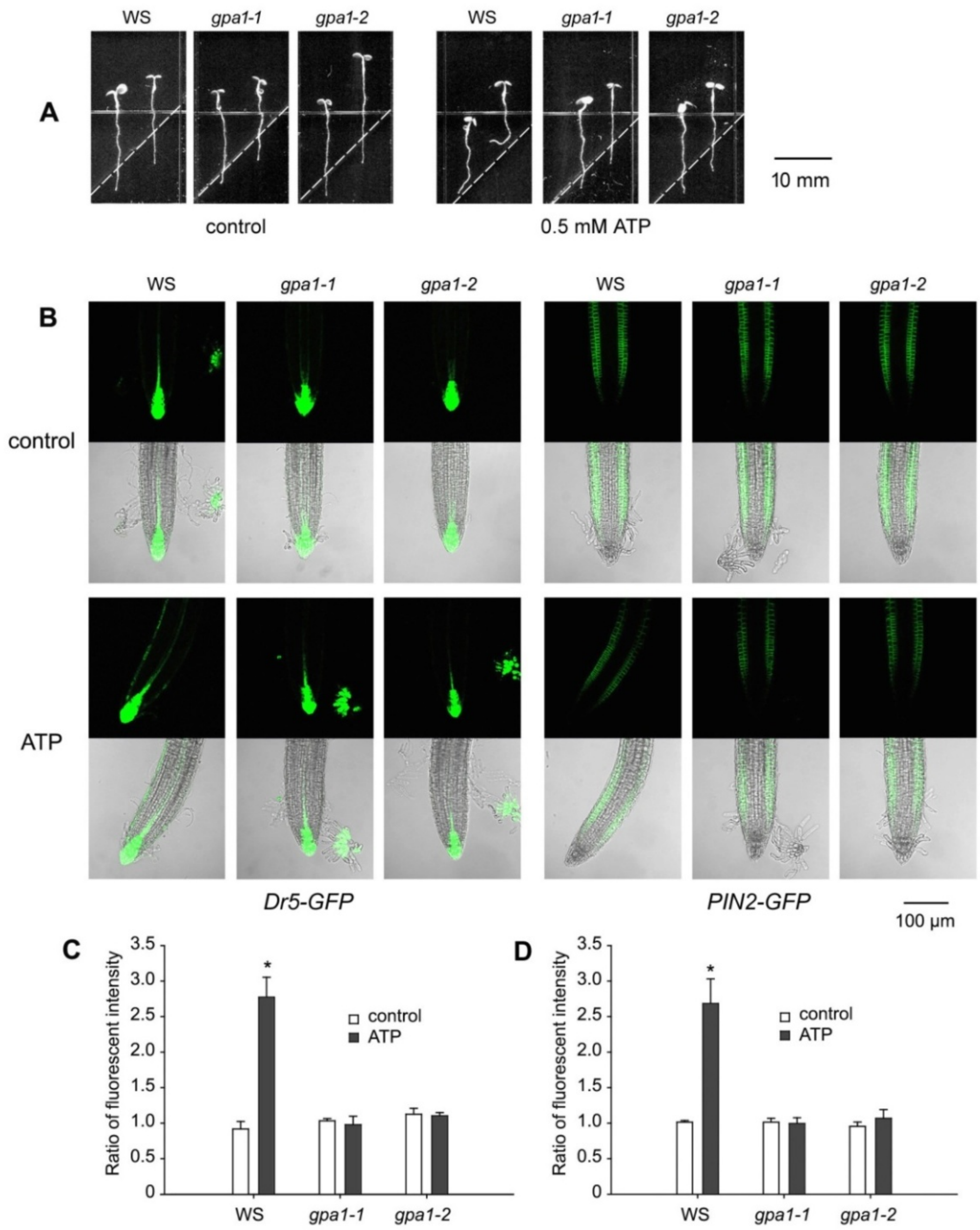

FIGURE S4│Response of roots to asymmetric ATP stimulation. **(A)** Seedlings of WS, Gα null mutants (*gpa1-1*, *gpa1-2*) were grown in untreated 1/2 MS medium for 4 days and transplanted onto diagonal jointed medium containing 0 (control) or 0.5 mM ATP (ATP) in the lower-right part, respectively. Seedlings were photographed 24 h after transplantation. In each photo, the dotted line marks the joint line of the two media. The scale bar is showed beside. **(B)** Fluorescence of DR5-GFP or PIN2-GFP in representative root tip cells. The scale bar is showed below. **(C,D)** Note the fluorescence intensity ratio (fluorescence intensity in left-side cells relative to that in right-side cells) of DR5-GFP **(C)** and PIN2-GFP **(D)**, respectively. In each experiment, fluorescence intensity in up to 10 roots was measured. Data from 3 replicates were calculated to get the mean ± SD. Student's *t*-test *p*-values: * $p<0.05$.

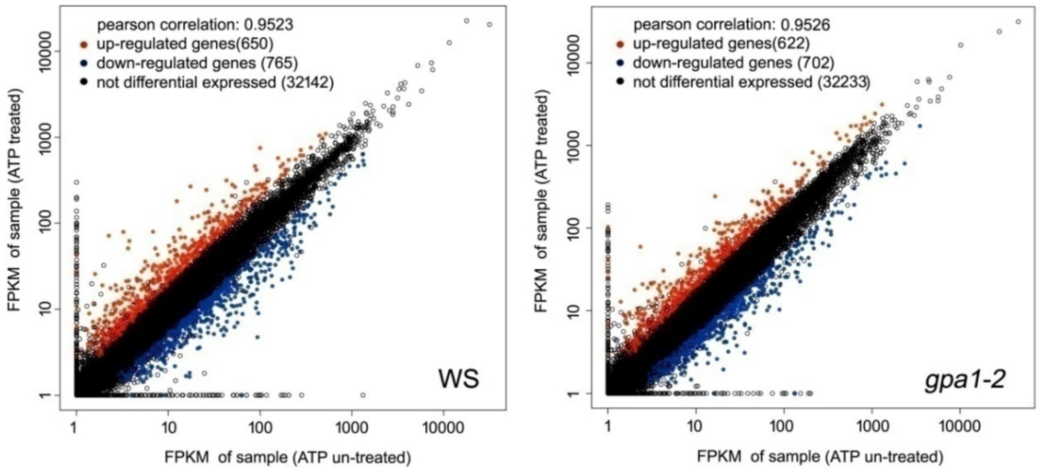

FIGURE S5│ ATP regulated gene expression in root cells. 5-day old seedlings were transplanted onto 0.5 mM ATP containing medium. After 60 min, RNA was extracted from roots and reverse-transcribed to cDNA. DNA microarray was performed to detect gene expression in WS and Gα null mutant (*gpa1-2*). Difference in gene expression of ATP treated vs untreated roots is showed in a scatter plot. The numbers of differential genes including up-regulated and down-regulated genes are summarized. **FPKM**: **F**ragments **P**er **K**ilobase of exon model per **M**illion mapped fragments.

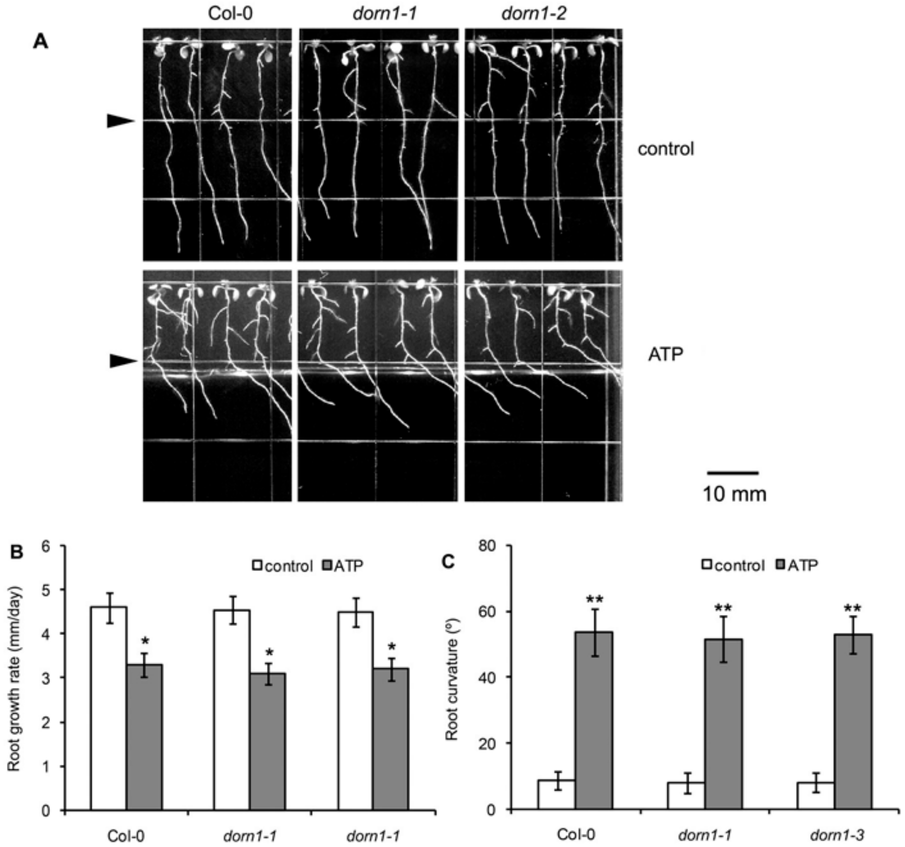

FIGURE S6│DRON1 is unlikely involved in eATP avoidance response of *Arabidopsis* roots. **(A)** The response of primary roots to eATP. In each photo series, the triangle marks the joint line of the two media. The scale bar is showed beside. **(B, C)** Note root growth rate **(B)** and root curvature **(C)**, respectively. Seedlings of Col-0, DORN1 null mutants (*dorn1-1*, *dorn1-3*) were grown in untreated 1/2 MS medium for 4 days and transplanted onto jointed medium containing 0 (control) or 0.5 mM ATP in the lower part, respectively. Seedlings were photographed 5 days after transplantation. In each experiment, primary root growth rate and curvature of at least 30 seedlings were measured, and data from 3 replicates were calculated to get the mean ± SD. Student's *t*-test *p*-values: * $p<0.05$, ** $p<0.01$.

Table S1. Primers for real-time qPCR analysis.

Gene Name	Gene locus	LP	RP
WAG1	At1g53700	TCTCAACCGCAACAACACCT	GCGAATCCGGTAGGGTTAGG
WAG2	At3g14370	CACATCCACCACTACCGACC	TCCGTCGGAGGAGAGAAGTT
Galactose oxidase	At3g27220	GTGACGGATGGACGGTATGT	GGAGCATACCTTGGAGCTGG
CRK40	At4g04570	GCAAGAGGCTACGACCAAGA	AGGAGGTTTAAGCTGGACGC
ATH7	At3g47790	GTTCGACCGATGGCTCTGAT	ACCCATGTGAAGAAGAGCGG
GH3.4	At1g59500	GCCGGGATTAGACAAAGGCA	GACGTCCTGAAGTAGTCGCT
OCT3	At1g16390	CCTCTGCACCTTACCCAACC	ACAGCCGAGGAAGAATGAGC
cyclin p3;1	At2g45080	AGGAACGAGAGAATCAGCCG	CGATGGACCGGCTTTGGTAT
ERF1	At3g23240	GGCGGAGAGAGTTCAAGAGT	TAACTTCACGGAGCGGTGAT
ERF114	At5g61890	GGTGGGTATGGGAGTGGTAC	AAGAAGAATCCCCAAAGCGC
CRK5	At3g50530	CAACGAACAATGAAGGCAAAA	GATCTCGCCGGAGTCTTCTT
ACTIN2	At3g18780	GGTAACATTGTGCTCAGTGGTGG	AACGACCTTAATCTTCATGCTGC

Table S2. Full name and function of some eATP induced proteins.

Abbreviation	Full name	Physiological function	Reference
WAG1, WAG2	Waved Aberrant Growth genes encoded protein	protein-serine/threonine kinases, involve in root waving growth	(Santner and Watson, 2006)
CRK5	CDPK (calcium dependent protein kinase)-RELATED KINASE 5	involve in PIN2 distribution and root gravitropism regulated by cytoplasmic Ca^{2+} signaling	(Rigo *et al.*, 2013)
CRK40	Cystein-rich receptor kinase 40	involve in pathogen defense and programmed cell death	(Wrzaczek *et al.*, 2010)
	galactose oxidase	involve in auxin-regulated cell wall synthesis	(Overvoorde *et al.*, 2005)
	Cyclin p3;1	involve in cell division as an activator of CDK4 (a cyclin-dependent protein kinase)	(Torres Acosta *et al.*, 2004)
ERF1, ERF114	ethylene responsive factors	involve in cell growth and defensive responses	(Muller and Munne-Bosch, 2015)
ATH7	ATP Binding Cassette transporter 2 homolog	involve in pathogen resistance, heavy metal detoxification and auxin transport	(Lefevre *et al.*, 2015)
GH3.4	auxin-responsive GH3 family protein	IAA-amido synthetase, involve in cellular auxin homeostasis	(Staswick *et al.*, 2005)
OCT3	organic cation/carnitine transporter 3	participate plant adaptation to environmental stress	(Kufner and Koch, 2008)

REFERENCES

Kufner, I., and Koch, W. (2008). Stress regulated members of the plant organic cation transporter family are localized to the vacuolar membrane. *BMC Res Notes* 1**,** 43. doi: 10.1186/1756-0500-1-43.

Lefevre, F., Baijot, A., and Boutry, M. (2015). Plant ABC transporters: time for biochemistry? *Biochem Soc Trans* 43(5)**,** 931-936. doi: 10.1042/BST20150108.

Muller, M., and Munne-Bosch, S. (2015). Ethylene Response Factors: A key regulatory hub in hormone and stress signaling. *Plant Physiol* 169(1)**,** 32-41. doi: 10.1104/pp.15.00677.

Overvoorde, P.J., Okushima, Y., Alonso, J.M., Chan, A., Chang, C., Ecker, J.R., *et al.* (2005). Functional genomic analysis of the AUXIN/INDOLE-3-ACETIC ACID gene family members in *Arabidopsis thaliana*. *Plant Cell* 17(12)**,** 3282-3300. doi: 10.1105/tpc.105.036723.

Rigo, G., Ayaydin, F., Tietz, O., Zsigmond, L., Kovacs, H., Pay, A., *et al.* (2013). Inactivation of plasma membrane-localized CDPK-RELATED KINASE5 decelerates PIN2 exocytosis and root gravitropic

response in *Arabidopsis*. *Plant Cell* 25(5)**,** 1592-1608. doi: 10.1105/tpc.113.110452.

Santner, A.A., and Watson, J.C. (2006). The WAG1 and WAG2 protein kinases negatively regulate root waving in *Arabidopsis*. *Plant J* 45(5)**,** 752-764. doi: 10.1111/j.1365-313X.2005.02641.x.

Staswick, P.E., Serban, B., Rowe, M., Tiryaki, I., Maldonado, M.T., Maldonado, M.C., *et al.* (2005). Characterization of an *Arabidopsis* enzyme family that conjugates amino acids to indole-3-acetic acid. *Plant Cell* 17(2)**,** 616-627. doi: 10.1105/tpc.104.026690.

Torres Acosta, J.A., de Almeida Engler, J., Raes, J., Magyar, Z., De Groodt, R., Inze, D., *et al.* (2004). Molecular characterization of *Arabidopsis* PHO80-like proteins, a novel class of CDKA;1-interacting cyclins. *Cell Mol Life Sci* 61(12)**,** 1485-1497. doi: 10.1007/s00018-004-4057-4.

Wrzaczek, M., Brosche, M., Salojarvi, J., Kangasjarvi, S., Idanheimo, N., Mersmann, S., *et al.* (2010). Transcriptional regulation of the CRK/DUF26 group of receptor-like protein kinases by ozone and plant hormones in *Arabidopsis*. *BMC Plant Biol* 10**,** 95. doi: 10.1186/1471-2229-10-95.
